# Transcytosis, Antitumor Activity and Toxicity of Staphylococcal Enterotoxin C2 as an Oral Administration Protein Drug

**DOI:** 10.3390/toxins8060185

**Published:** 2016-06-16

**Authors:** Wenbin Zhao, Yangyang Li, Wenhui Liu, Ding Ding, Yingchun Xu, Liqiang Pan, Shuqing Chen

**Affiliations:** 1 College of Pharmaceutical Science, Zhejiang University, Hangzhou 310058, China; pharmacy_zwb@zju.edu.cn (W.Z.); leakey0216@126.com (Y.L.); liuwenhui1990@163.com (W.L.); ycxu66@163.com (Y.X.); 2 Hisun Pharma (Hangzhou). CO., LTD., Xialian Village, Xukou Town, Fuyang, Hangzhou 311404, China; dingding@hisunpharm.com

**Keywords:** staphylococcal enterotoxin C2, superantigen, oral administration, antitumor activity, toxicity

## Abstract

Staphylococcal enterotoxin C2 (SEC2) is a classical superantigen (SAg), which can tremendously activate T lymphocytes at very low dosage, thus exerting its powerful antitumor activity. As an intravenous protein drug and a bacterial toxin, SEC2 has some limitations including poor patient compliance and toxic side effects. In this research, we devoted our attention to studying the antitumor activity and toxicity of SEC2 as a potential oral administration protein drug. We proved that His-tagged SEC2 (SEC2-His) could undergo facilitated transcytosis on human colon adenocarcinoma (Caco-2) cells and SEC2-His was detected in the blood of rats after oral administration. Furthermore, oral SEC2-His caused massive cytokine release and immune cell enrichment around tumor tissue, leading to inhibition of tumor growth *in vivo*. Meanwhile, although SEC2-His was dosed up to 32 mg/kg in mice, no significant toxicity was observed. These data showed that SEC2 can cross the intestinal epithelium in an immunologically integral form, maintaining antitumor activity but with reduced systemic toxicity. Therefore, these results may have implications for developing SEC2 as an oral administration protein drug.

## 1. Introduction

Staphylococcal enterotoxins (SEs), secreted by *Staphylococcus aureus*, are classical models of superantigens (SAgs) [1]. SEs can directly bind to the antigenic groove of major histocompatibility complex class II (MHC II) molecules and the beta-chain variable region (Vβ) of the T-cell receptor in the complete form, then stimulate numerous T-cells for proliferation and to release massive amounts of cytokines like interleukin (IL)-2, IL-4, tumor necrosis factor (TNF) and interferon-γ (IFN-γ) [2,3,4]. What’s more, SEs direct cytotoxic T lymphocyte (CTL) mediated cytotoxicity against target cells [5]. Therefore, SEs could show powerful antitumor effects *in vitro* and *in vivo*. SEs include SEA, SEB, SEC, SED, SEE, SEG, SEH, SEI and SEU [1,6]. As a subtype of SEC [7], SEC2 has been used clinically as a supplementary therapeutic agent for tumor treatment for many years. In China, staphylococci injection is widely used in cancer therapy, and the main effective component is claimed to be SEC2 [8]. Many clinical reports have confirmed that staphylococci injection has obvious immunomodulatory effects [9,10]. The injection could strongly increase patients’ CD4^+^ T-cell numbers and significantly reduce the side effects related to chemotherapy, which provides patients with short-term efficacy and long-term survival benefit [9,10].

Although staphylococci injection has shown efficacy in cancer therapy, it causes severe side effects because of direct intravenous dosing and its complex constituents derived from the preparation method, *i.e.*, filtrate of fermentation broth of *Staphylococcus aureus* [11,12]. The purity of staphylococci injection could be solved using independent expression of its active ingredient SEC2. However, SEs could cause emesis, fever, and other side effects due to their toxic activity, which limits their clinical application [1,13]. While oral dosing would be a potential viable strategy to reduce potential side effects resulting from intravenous injection, there are real challenges in developing protein drugs for oral administration. The clinical development of oral protein drugs has been restricted due to their poor membrane permeation and instability against enzymatic degradation leading to limited absorption in the gastrointestinal tract. Therefore, injection is preferred for protein drug administration [14,15]. Hamad *et al.* [16] have proved the human intestinal permeability of SEA and SEB by Caco-2 permeability assay, and dose-dependent transcytosis of SEB and SEA was determined in the blood, indicating that SEs might cross the epithelium in an immunologically intact form and interact with the host immune system. In addition, it has been demonstrated that oral SEC2 showed no obvious toxicity when given to monkeys [13]. Therefore, we evaluated the *in vivo* efficacy of oral SEC2 as a potential antitumor agent, followed by a toxicity study. Both *in vitro* and *in vivo* transcytosis of SEC2 was determined by using the Caco-2 permeability assay and blood test of Sprague-Dawley (SD) rats after oral administration of SEC2.

## 2. Results

### 2.1. Transcytosis of SEC2-His through Caco-2 Monolayers

**Figure 1:**
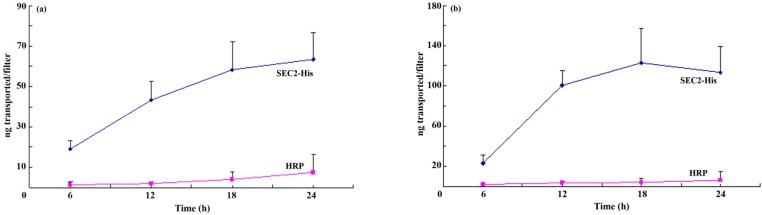
His-tagged staphylococcal enterotoxin C2 (SEC2-His) transcytosis by Caco-2 cells is bidirectional and time dependent for 24 h. (**a**) Transcytosis of SEC2-His by Caco-2 cells from apical to basolateral surfaces. (**b**) Transcytosis of SEC2-His by Caco-2 cells from basolateral to apical surfaces. Compared with Horseradish peroxidase (HRP), the rate and the total quantity of transcytosis SEC2-His were both significantly higher. The transcytosis rate of SEC2-His from apical to basolateral surfaces was slightly lower than basolateral to apical transcytosis. The results were expressed as mean ± S.D (*n* = 3).

We prepared two chambered cultures separated by a monolayer of confluent Caco-2 cells as described in Methods and assessed transepithelial electrical resistance of monolayer Caco-2 cells at day 3, 12 and 21. The transepithelial electrical resistance of monolayer Caco-2 cells reached 900 Ω/cm^2^ at day 21, which complied with the requirements of the follow-up experiments. To study whether SEC2-His could be transported by Caco-2 cells specifically, we used Horseradish peroxidase (HRP) as an internal control to distinguish between specific transcytosis and nonspecific transport such as paracellular diffusion with water. It was demonstrated that SEC2-His could cross Caco-2 cell monolayers bidirectionally and the rate of its transcytosis was linear for 24 h. Compared with HRP, the rate and the total quantity of transcytosis SEC2-His were both significantly higher. These results also showed that the transcytosis of SEC2-His from basolateral to apical surfaces by Caco-2 cells could be saturated in 18 h. However, this phenomenon was not observed in forward transcytosis even in 24 h. In general, the transcytosis rate of SEC2-His from apical to basolateral surfaces was slightly lower than basolateral to apical transcytosis (Figure 1).

**Figure 2:**
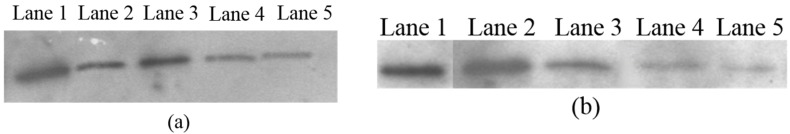
Western-Blotting analysis of transcytosis SEC2-His. (**a**) Transcytosis of SEC2-His by Caco-2 cells from apical to basolateral surfaces. Lane 1: standard SEC2-His; lane 2: 24-h sample; lane 3: 18-h sample; lane 4: 12-h sample; lane 5: 6-h sample; (**b**) Transcytosis of SEC2-His by Caco-2 cells from basolateral to apical surfaces. Lane 1: standard SEC2-His; lane 2: 24-h sample; lane 3: 18-h sample; lane 4: 12-h sample; lane 5: 6-h sample.

To see if the transcytosed SEC2-His maintained its integrity, samples from different time points were analyzed by western-blotting (Figure 2). The molecular weight of SEC2-His (about 28 kDa) in all samples was consistent with standard SEC2-His, indicating that SEC2-His could cross monolayer Caco-2 cells in the form of intact molecules. Additionally, the amount of transcytosed SEC2-His was positively correlated with time, which was consistent with the results of Figure 1. The above results showed that the transcellular transport of SEC2-His is specific transcytosis by Caco-2 cells instead of non-specific transport such as free diffusion.

### 2.2. Pharmacokinetics of SEC2-His in Rat

**Figure 3:**
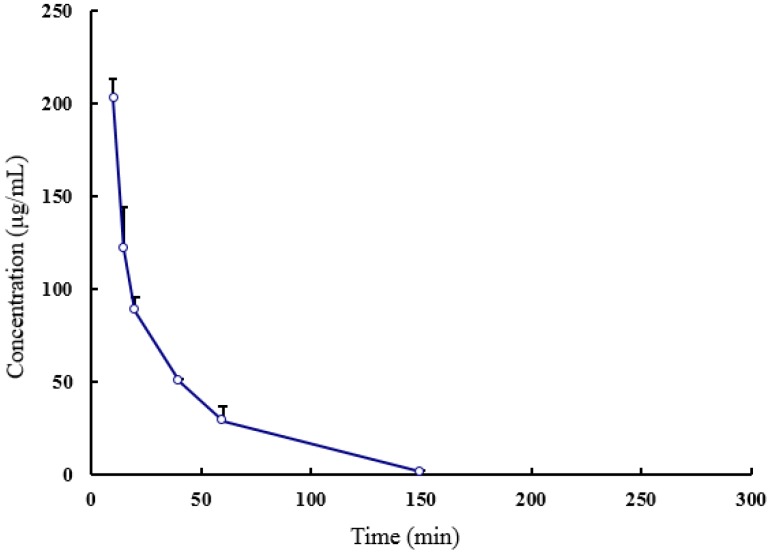
Pharmacokinetic curve of SEC2-His in rats. The results were expressed as mean ± S.D (*n* = 3).

A quantity of 2.4 mg SEC2-His was injected into three SD rats via tail vein, and we collected sera at 0 h, 5 min, 10 min, 15 min, 20 min, 40 min, 1 h, and 2.5 h. Then the concentration of SEC2-His in serum was determined through sandwich enzyme-linked immunosorbent assay (ELISA) and we analyzed the pharmacokinetic parameters of serum SEC2-His concentration profiles by non-compartment model analysis in DAS 2.0 software. The result showed that the average half-life of SEC2-His in rat is about 20 min (Table 1), and, 2.5 h after injection, the concentration of SEC2-His in serum was below the detection limit (2 ng/mL), which meant that almost all SEC2-His was metabolized (Figure 3).

### 2.3. Oral Administration of SEC2-His to Rats

We collected 0-h and 2-h serum samples after oral administration of 25 mg SEC2-His to three rats and detected SEC2-His by sandwich ELISA. The OD value of 2-h serum samples in each rat was at least 2.5 times more than the OD value of 0-h serum samples (data not shown). These results showed that SEC-His could be absorbed following oral administration in rats.

### 2.4. Antitumor Activity of SEC2-His in ICR Mice

Antitumor activity of SEC2-His was studied using the mouse hepatoma cell line (H22) in an ICR (Institute of Cancer Research) mouse liver cancer xenograft model. His-tagged Sortase A (Sortase A-His), the molecular weight of which is similar to SEC2-His, was used as a negative control. The antitumor agent cyclophosphamide (CTX) was used as a positive control. After treatment of xenografted mice with SEC2-His for nine days, we observed significant differences of tumor size among test groups and the negative control group, and then tumors were isolated and weighed. As shown in Table 2, the inhibition rate of treatment groups respectively was 50.82% (32 mg/kg SEC2-His), 44.74% (16 mg/kg SEC2-His), and 25.33% (6.4 mg/kg SEC2-His). Among them, the inhibition rates of high dose and the middle dose groups were significantly different with the saline group by t test. However, we observed that the tumor weight of the Sortase A-His group was approximately the same as the saline group, which revealed that Sortase A-His did not have antitumor activity, and the His-tag was not the antitumor active site of SEC2-His.

**Figure 4:**
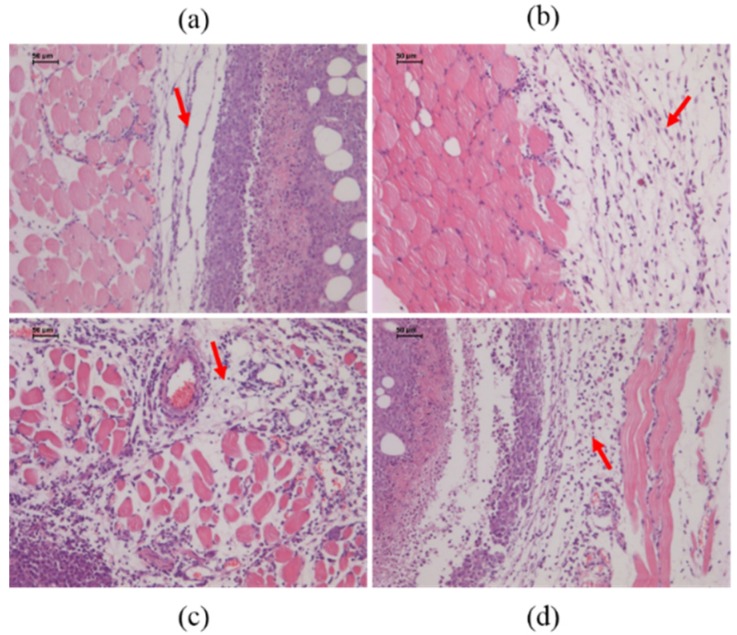
H & E staining of tumor tissues. The areas indicated by red arrows represent the enrichment of immune cells in: (**a**) the saline group; (**b**) the SEC2-His (6.4 mg/kg) group; (**c**) the SEC2-His (16 mg/kg) group; (**d**) the SEC2-His (32 mg/kg) group. In the saline group, few immune cells were detected near the tumor tissue, while more immune cells were enriched near tumor tissues of treatment groups and cell numbers increased in a dose-dependent manner. Magnification of tumor tissues was 100×.

In addition, we tested the infiltration of immune cells to the tumor by H & E (Hematoxylin and Eosin) staining. Compared with the saline group, the treatment groups were found to have more immune cells enriched in para-carcinoma tissue in a dose-dependent manner (Figure 4). All results showed that oral administration of SEC2-His in ICR mice resulted in effective antitumor activity *in vivo*.

### 2.5. Cytokine Assays of SEC2-His Fed ICR Mice

**Figure 5:**
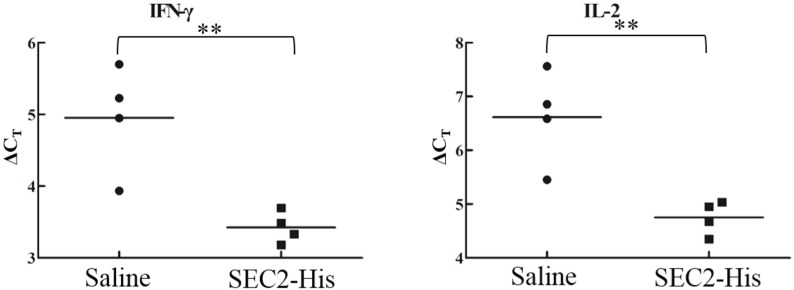

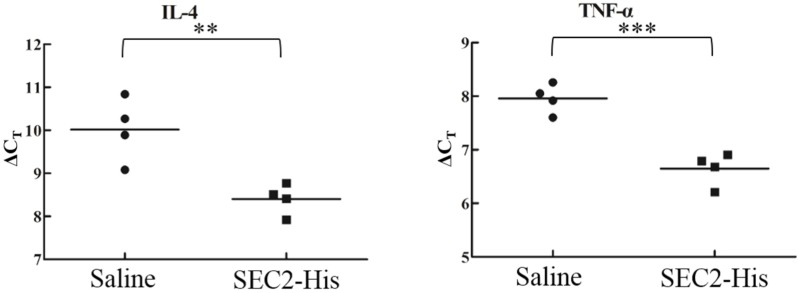
The expression level of interferon (IFN)-γ, interleukin (IL)-2, IL-4 and tumor necrosis factor (TNF)-α. Total splenic mRNA was extracted from all spleens to analyze the expression level of cytokine transcripts by real-time PCR after 24 h of administration of SEC2-His or saline. * *p* < 0.05, ** *p* < 0.01, *** *p* < 0.001.

Eight ICR mice were randomly divided into two groups and given by gavage SEC2-His (32 mg/kg) or saline separately. After 24 h we extracted spleen total mRNA to detect the levels of IL-2, IL-4, TNF-α and IFN-γ by Real-time PCR. The results showed *C_t_*_(target gene)_ values in all treatment groups were more than *C_t_*_(β-actin)_, which means that the expression level of β-actin is higher than all target genes. As Δ*C_t_* = *C_t_*_(target gene)_ − *C_t_*_(β-actin)_, the larger the Δ*C_t_* value was, the lower the relative expression of target genes. As Figure 5 shows, the Δ*C_t_* values of all the target genes from the SEC2-His group are smaller than the saline group. So we could make a conclusion that compared with the saline group, the mRNA expression levels of these four cytokines were significantly up-regulated after oral SEC2-His administration in mice. In addition, the average Δ*C_t_* (AΔ*C_t_*) of different target genes were marked in Figure 5 (as shown by the “—”) and the fold changes of mRNA expression levels of IL-2, IL-4, TNF-α and IFN-γ due to treatment with SEC2-His were 3.634, 3.069, 2.480, and 2.886 respectively.

### 2.6. Toxicity Evaluation of SEC2-His Fed Mice

**Figure 6:**
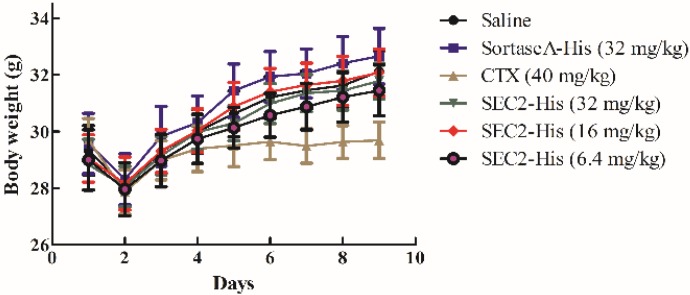
Body weight of mice. All samples were weighed every day, and the results were expressed as mean ± S.D. from 10 different mice.

**Figure 7:**
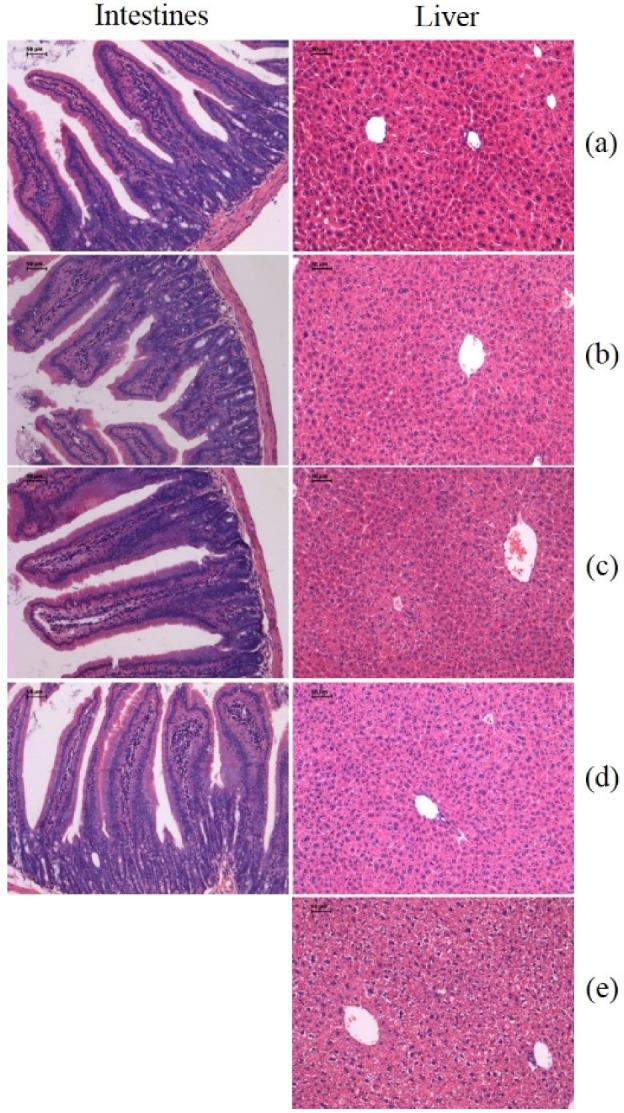
Histologic investigations of intestines and livers for toxicity evaluation. One mouse was randomly picked from each group, and tissues were examined after H & E staining: (**a**) the saline group; (**b**) the SEC2-His (6.4 mg/kg) group; (**c**) the SEC2-His (16 mg/kg) group; (**d**) the SEC2-His (32 mg/kg) group; (**e**) the positive group. In the positive group, the ultrastructural changes showed hydropic degeneration in liver, while the other groups were assessed as normal. Magnification of intestines and livers was 100× and 400× respectively.

**Figure 8:**
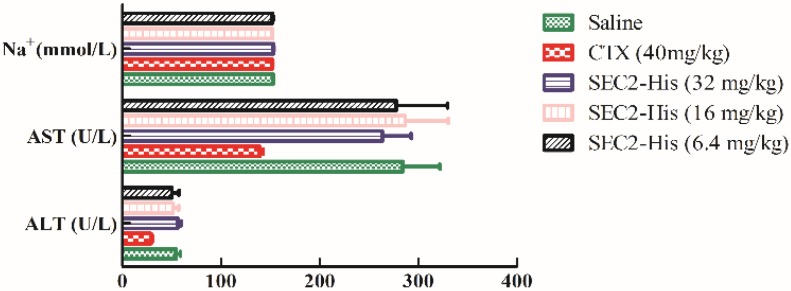
Analysis of alanine transaminase (ALT), aspartate transaminase (AST) and Na^+^ for toxicity evaluation. The sera extracted from ICR (Institute of Cancer Research) mice were analyzed by Cobas C 31 (Roche, Basel, Switzerland) to detect the levels of ALT, AST and Na^+^. Results are expressed as mean ± S.D. from 3 different mice.

We weighed all groups of mice and observed the defecation pattern. The results showed the weight of all groups increased continually, of which the growth rate of treatment groups and the saline group were approximately the same, but the CTX group’s growth rate was distinctly lower than others (Figure 6, Table 2). Meanwhile we did not observe abnormal defecation pattern in any of the groups. Liver and intestinal tissues were stained by H & E to further assay the toxicity of oral SEC2-His. The treatment and saline groups did not show toxicity of liver and intestinal tissues through morphological examination, however, the positive group showed significant liver toxicity (Figure 7). In the meantime, we tested the levels of aspartate transaminase (AST), alanine transaminase (ALT) and Na^+^ in mice sera. The level of AST, ALT and Na^+^ in all groups was in the normal range except that the positive group’s AST and ALT were lower than normal values (Figure 8). Therefore, we proved that oral SEC2-His had no obvious toxicity in mice.

## 3. Discussion

As a superantigen, SEC2 can combine in integrated molecular structure with MHC II molecules on antigen presenting cells (APCs) to form the complex. Then the complex binds to a particular region on TCR Vβ [17], which intensely induces T-cell activation and proliferation [18]. Through superantigen dependent cytotoxic effect and cytokine production, SEC2 can trigger the killing of tumor cells, showing good antitumor activity [19]. Nowadays, staphylococci injection, whose major effective component is SEC2, is widely applied as a biological response modifier (BRM) in tumor therapy. The injections have been confirmed to strongly increase CD4^+^ T-cell numbers and percentage of Nature Killer (NK) cells in the treatment patients, which provides them with short-term efficacy and long-term survival benefit [9,10]. However, the complex composition and high toxicity were the major shortcomings of clinical application of staphylococci injection. What’s more, our present study showed that the half-life of SEC2 (active component of staphylococci injection) in rats was only about 20 min. Therefore, poor pharmacokinetics (PK) of SEC2 may lead to frequent injections for patients to achieve certain efficacy, further conferring pain and inconvenience to patients. Intriguingly, it has been shown that SEs such as SEA and SEB could cross the epithelium with complete biological activity [16], which provides a possibility to address all the above issues. Because oral administration of SEC2 alone would be much more convenient, and could largely reduce the possible side effects caused by impurities from staphylococci injection and the risk of fast distribution in blood. Therefore, we have investigated whether SEC2 could be absorbed and maintain its antitumor activity after oral administration. The ability of SEC2-His to transcytoses *in vitro* was tested and we found that SEC2-His could cross Caco-2 cells bidirectionally. The rate and total quantity of transcytosed SEC2-His were significantly higher than HRP, suggesting the transcellular transport of SEC2-His was specific transcytosis by Caco-2 cells. Meanwhile, we measured the oral administration of SEC2-His *in vivo* and found it can be absorbed. Although the mechanism of SEC2 transcytosis has not been extensively studied, previous studies have shown that some cells, like B-cells, mast cells and intestinal epithelia, have non-MHC receptors that can bind to SEs [20,21,22]. We speculate that SEC2 might be transported through a functional domain interacting with a specific receptor, which is distributed on both the basolateral and apical sides of intestinal epithelial cells. Since the transcytosis rate of SEC2-His from apical side to basolateral side was slightly lower than basolateral to apical, there might be less receptors on the apical side than basolateral side of Caco-2 cells. In general, most proteins cannot be absorbed by the intestines in an intact form, and the transcytosis mechanism of oral SEs could probably be applied to develop other oral protein drugs.

We studied the antitumor activities of SEC2-His *in vivo*, and found oral SEC2-His could cause the up-regulated expression of numerous cytokine transcripts and stimulate proliferation and enrichment of immune cells around tumor tissue to inhibit tumor growth, which might be the same antitumor mechanism as injected SEs [23,24]. Interestingly, there was no enrichment of immune cells found in intestinal tissue through H & E staining. Therefore, SEC2 might cross the intestinal epithelium followed by stimulation of the lymphatic system to exert antitumor activity, instead of directly stimulating intestinal lymphocytes. Studies showed that there were different ways of combining SEs with MHC II molecules [25]. The more stable the interaction was; the less SEs were needed to exert the same amount of biological effect [26,27]. As the ability of SEs combining with human MHC II molecules was much stronger than with mice MHC II molecules, the requirement of SEs for activation of human peripheral blood mononuclear cells was far less than mice [26,27]. Therefore, we speculate less oral SEC2 will be needed for humans than mice while exerting a better antitumor activity.

As reported, SEs could directly affect intestinal epithelial cells and the vagus nerve, causing vomiting and diarrhea [1]. Therefore, some researchers have successfully reduced the toxic side effects of SE treatment through site-specific mutagenesis [19,28]. However, we found no obvious damage in intestine and liver in our study and the level of Na^+^, ALT and AST were approximately the same among treatment groups and saline group. These interesting results imply the promising application of oral SEC2 in antitumor immunotherapy.

## 4. Materials and Methods

### 4.1. Cell lines, Drugs, Animals, Antibodies

Caco-2 cells, obtained from American Type Culture Collection (ATCC), were generated and maintained in Dulbecco’s Modified Eagle Medium (DMEM, Gibco, Grand Island, CA, USA) with 10% Fetal Bovine Serum (FBS, Gibco, Grand Island, CA, USA) and 1% non-essential amino acids (Gibco, Grand Island, CA, USA). H22 cells, kindly provided by the Laboratory of Tumor and Endocrine Pharmacology, College of Pharmaceutical Sciences, Zhejiang University (Hangzhou, China), were generated and maintained in RPMI Medium 1640 (RPMI-1640, Gibco, Grand Island, CA, USA) with 10% FBS. SEC2-His was purified and preserved by our laboratory [29]. All animals were purchased from Slaccas (Shanghai, China) and housed in an air-conditioned room, with temperatures of 23 ± 2 °C, relative humidity 50%–60%, and controlled illumination of a 12 h light-dark cycle at the Laboratory Animal Center of Zhejiang University. All procedures described in this study were reviewed and approved by the Ethics Committee for the Use of Animals at Zhejiang University, China. Rabbit anti-SEC2 polyclonal antibody was produced and preserved by our laboratory [30]. Biotinylated anti-SEC2 monoclonal antibody was purchased from Toxin Technology, Inc. Other antibodies were purchased from Beyotime (Shanghai, China).

### 4.2. Transcytosis of SEC2-His

Caco-2 cells, in logarithmic growth phase, were suspended in cell culture medium at 1 × 10^5^ cells/mL. Cell suspensions were added to the 12-well Transwell™ plates (Corning, Corning City, NY, USA). Medium was replaced every other day until confluent monolayers with tight junctions developed as assessed by transepithelial electrical resistance of monolayers that was measured with a Millicell-ERS voltohmmeter (Millipore Corp., Billerica, MA, USA). Only monolayers with transepithelial electrical resistance of ≥900 Ω/cm^2^ after correction for intrinsic value of empty filter were used to test the ability of Caco-2 cells to transcytose SEC2-His as follows: HRP (40 kDa) was added as an internal control for nonspecific transcytosis because its molecular weight is close to SEC2-His. Medium was removed and the selected filters were washed twice using HBSS (supplementary material). After washing, fresh HBSS containing SEC2-His (7.5 μg/mL) or HRP (10 μg/mL) were added to either the apical (500 μL) or basolateral (1.5 mL) chamber followed by incubation in a horizontal shaker (30 rpm) at 37 °C for 6, 12, 18 or 24 h. After incubation, apical or basolateral medium was collected depending on whether SEC2-His or HRP were added to the basolateral or apical medium. The concentrations of SEC2-His or HRP in collected samples were determined by sandwich ELISA using rabbit anti-SEC2 polyclonal antibody or colorimetric method (Supplementary Material). The integrity of SEC2-His was assayed by western blotting (Supplementary Material).

### 4.3. Pharmacokinetics of SEC2-His

Three male SD rats, weighing about 200 g, were injected via tail vein with 2.4 mg SEC2-His. Blood samples were collected before administration and at different time points after administration, and then incubated at 37 °C for 30 min followed by centrifugation (2000 rcf, 30 min) to obtain sera. The concentrations of SEC2-His in sera were determined by sandwich ELISA using biotinylated anti-SEC2 monoclonal antibody (Supplementary Material). The linear range of the sigmoidal titration curve of standard SEC2-His was used to calculate the concentrations of SEC2-His in blood samples. Pharmacokinetic parameters were obtained from serum SEC2-His concentration profiles using non-compartment model analysis in DAS 2.0 software (Mathematical Pharmacology Professional Committee of China, Shanghai, China) [31].

### 4.4. Oral Administration of SEC2-His to Rats

Three male SD rats, weighing about 200 g, were treated with 25 mg SEC2-His during gavage feeding. Blood samples were collected before administration and 2 h later after administration, then sera were obtained as described in the previous protocol. SEC2-His in sera was detected by sandwich ELISA using rabbit anti-SEC2 polyclonal antibody (Supplementary Material).

### 4.5. Antitumor Activity in Vivo of SEC2-His

Sixty male ICR mice, age about 8 weeks and weight about 29 g, were used in this experiment. To generate tumor-bearing ICR mice, freshly harvested H22 cells (2 × 10^5^ cells per mouse) were inoculated subcutaneously in the right flank of ICR mice. The day after inoculation, sixty mice were randomly divided into 6 groups and treated with saline, Sortase A-His (32 mg/kg), SEC2-His (32 mg/kg, 16 mg/kg or 6.4 mg/kg) during gavage feeding and CTX (40 mg/kg) during intraperitoneal injection per day for 9 days.

The day after last administration, tumors were isolated, weighed, fixed with 10% neutral-buffered formalin and paraffin-embedded. Then sections were cut from tumor tissue and stained by H & E. Immune cell infiltration in tumors were evaluated through morphological examination. Tumor growth inhibition rate was measured by the following equation: Inhibition rate (%) = (Tumor weight _saline_ − Tumor weight _test group_)/Tumor weight _saline_ × 100.

### 4.6. Cytokines Assay

Ten male ICR mice as described above were randomly divided into two groups and orally administered SEC2-His (32 mg/kg) or saline. 24 h later, total spleen cellular mRNAs were extracted from ten mice by AxyPrep™ Multisource Total RNA Miniprep Kit (Axygen Biosciences, Silicon Valley, CA, USA) and then transcribed into cDNAs using PrimeScript™ RT Master Mix (Takara Bio, Japan). The mRNA levels of IL-2, IL-4, TNF-α, IFN-γ and β-actin (endogenous housekeeping gene) were measured by Real-time PCR on StepOne Real-time PCR system with SYBR Premix Ex Taq II Kit (Takara Bio). Primers used are listed in Table 3. Each reaction buffer was carried out in 10 μL volume containing 5.0 μL SYBR Premix Ex Taq II, 0.2 μL ROX Reference, 0.4 μL forward and reverse primers each, 1.0 μL diluted template cDNA and 3.0 μL ddH_2_O. The amplification steps included a preincubation at 95 °C for 30 s, followed by 40 cycles of denaturing at 95 °C for 5 s, annealing at 52.5 to 55.5 °C for 30 s, elongation at 72 °C for 30 s. The specificity of amplicons was measured and verified by analyzing the melting curve after 40 cycles. Gene expression levels were measured by the following equation: Δ*C_t_* = *C_t_*_(target gene)_ − *C_t_*_(β-actin)_, Δ(AΔ*C_t_*) = AΔ*C_t_*_saline_ − AΔ*C_t_*_SEC2-His_, fold change due to treatment = 2^Δ(AΔC*t*)^.

### 4.7. Toxicity Evaluation of SEC2-His

Mice used to assess antitumor activity *in vivo* of SEC2-His were used to evaluate toxicity of SEC2-His as well. Body weight and defecation were monitored every day after first administration. Body weight growth rate was measured by the following equation: weight growth rate = (body weight _last day_ − body weight _first day_)/body weight _first day_ × 100. The day after last administration, liver and intestinal tissues were obtained, fixed with 10% neutral-buffered formalin, paraffin-embedded and stained by H & E according to the steps mentioned above, then acute toxicity of the two tissues were studied through morphological examination. Sera were obtained to be analyzed by Cobas C 31 (Roche) to determine the levels of AST, ALT and Na^+^.

### 4.8. Statistical Analysis

Statistical analysis was performed using Student’s *t*-test. *p*-value less than 0.05 was considered statistically significant. Results were presented as the Mean ± S.D.

## Figures and Tables

**Table 1:** Pharmacokinetic parameters of SEC2-His in rats at 2.4 mg/rat. AUC_inf_^1^: Area under the curve from zero to infinity; *t*_1/2_^2^: Half-life; CL^3^: Clearance.

Pharmacokinetic Parameters	Rat 1	Rat 2	Rat 3	Mean	SD
AUC_inf_^1^ (mg/L/min)	7781.463	8426.116	8237.809	8148.463	331.484
*t*_1/2_^2^ (min)	15.661	25.623	18.321	19.868	5.158
CL^3^ (L/min/kg)	0.002	0.001	0.001	0.001	0.001

**Table 2:** *In vivo* antitumor activities of SEC2-His. * *p* < 0.05, ** *p* < 0.01, *** *p* < 0.001.

Groups	Weight Growth Rate	Tumor Weight	Tumor Growth Inhibition Rate
Saline	8.675 ± 1.781	0.6080 ± 0.1012	/
Sortase A-His (32 mg/kg)	9.305 ± 2.491	0.6120 ± 0.1215	−0.65
SEC2-His (32 mg/kg)	9.206 ± 2.594	0.2990 ± 0.06681	50.82 *
SEC2-His (16 mg/kg)	9.387 ± 1.585	0.3360 ± 0.08415	44.74 *
SEC2-His (6.4 mg/kg)	7.816 ± 2.068	0.4540 ± 0.1046	25.33
CTX (40 mg/kg)	−0.041 ± 1.512 **	0	100 ***

**Table 3:** Primers for real-time PCR.

Gene	GenBank ID	Primer (5′ to 3′)	Tm (°C)	Product Size (bp)
IL-2	NM_008366.3	GCGGCATGTTCTGGATTTGACT (forward)	52.5	136
		CTCATCATCGAATTGGCACTCA (reverse)		
IL-4	M25892.1	CTCGAATGTACCAGGAGCCAT (forward)	55.5	133
		TTGCTGTGAGGACGTTTGG (reverse)		
TNF-α	BC117057.1	TGAGGTCAATCTGCCCAAGTA (forward)	55.5	268
		AGGTCACTGTCCCAGCATCT (reverse)		
IFN-γ	NM_008337.3	AACTCAAGTGGCATAGATGTGGAAG (forward)	54.1	256
		TGTTGACCTCAAACTTGGCAATAC (reverse)		
β-actin	NM_007393.3	AGAGGGAAATCGTGCGTGAC (forward)	55.5	204
		CACAGGATTCCATACCCAAG (reverse)		

## Supplementary Materials

The following are available online at www.mdpi.com/2072-6651/8/6/185/s1.
