# Supplementary material for: Transcytosis, Antitumor Activity and Toxicity of Staphylococcal Enterotoxin C2 as an Oral Administration Protein Drug
Source: Toxins (Basel). 2016 Jun 16;8(6):185. doi: 10.3390/toxins8060185 (PMC4926151; doi:10.3390/toxins8060185)
Supplement: Supplementary file 1 [file toxins-08-00185-s001.pdf]

Supplementary Materials: Transcytosis, Antitumor Activity and Toxicity of Staphylococcal Enterotoxin C2 as an Oral Administration Protein Drug

Wenbin Zhao, Yangyang Li, Wenhui Liu, Ding Ding, Yingchun Xu, Liqiang Pan and Shuqing Chen

Formula of HBSS: A buffer: 16 g NaCl, 0.8 g KCl, 0.2 g $MgSO_4.7H_2O$, 0.2 g $MgCl_2.6H_2O$, 0.28 g $CaCl_2$ dissolved in 1 L ultra-pure water and adjusted pH to 7.4.B buffer: 0.3 g $Na_2HPO_4.12H_2O$, 0.12 g KH_2PO_4, 2 g glucose dissolved in 1 L ultra-pure water and adjusted pH to 7.4. A buffer and B buffer were sterilized and mixed in equal volumes.

Sandwich ELISA: 96-well plates were coated by adding 100 μL of anti-his monoclonal antibody (500 μg/mL in carbonate-bicarbonate buffer, pH 9.6) overnight at 4 °C. After that, the plates were blocked with defatted milk in PBST buffer (PBS, pH 7.4, containing 0.05% of Tween-20) for 2 h at 37 °C. After blocking, each well was incubated with standard SEC2-His or samples that were diluted to serial fine concentration by blocking buffer for 2 h at 37 °C. 100 μL of rabbit anti-SEC2 polyclonal antibody or biotinylated anti-SEC2 monoclonal antibody (1 μg/mL in blocking buffer) was added to each well for 2 h at 37 °C, following by incubation with 100 μL HRP-tagged antibody or streptavidin (diluted 1:2000 in blocking buffer) for 1 h at 37 °C. 100 μL of tetramethylbenzidine (TMB, 75 μg/mL) substrate solution was added to each well and then incubated for 30 min at 37 °C. After that, 100 μL of stop solution (2 M H_2SO_4) was added to each well. Finally, the absorbance was measured at 450 nm in an ELISA plate reader.

Colorimetric method: Standard HRP solution or samples were diluted to serial fine concentration and added into 96-well plates. 50 μL of TMB (75 μg/mL) was added to each well and then incubated for 5 min at 37 °C. After that, 50 μL of stop solution (2 M H_2SO_4) was added to each well. Finally, the absorbance was measured at 450 nm in an ELISA plate reader.

Western-blotting: Standard SEC2-His or samples were separated on 12% SDS-polyacrylamide gel using a Protean Minigel apparatus (BioRad, Hercules, CA, USA), and proteins were transferred to a polyvinyldifluoride membrane (Millipore, Billerica, MA, USA). After blocking the membrane with defatted milk in PBST buffer (PBS, pH 7.4, containing 0.1% of Tween-20) at 4 °C overnight, the membrane was incubated with anti-his monoclonal antibody at 37 °C for 2 h. After that, membrane was washed 4 times using PBST buffer and then incubated with HRP tagged goat anti mouse antibody at 37 °C for 2 h followed by washing membrane 4 times. At last, membrane was incubated with ECL reagent (Biological Industries, Nahariya, Israel) and light emission detected by exposing the membrane to an X-ray film (Kodak, Rochester, NY, USA).
